# Global conservation prioritization for the Orchidaceae

**DOI:** 10.1038/s41598-023-30177-y

**Published:** 2023-04-25

**Authors:** Pati Vitt, Amanda Taylor, Demetra Rakosy, Holger Kreft, Abby Meyer, Patrick Weigelt, Tiffany M. Knight

**Affiliations:** 1 Chicago Botanic Garden, 1000 Lake Cook Road, Glencoe, IL 60022 USA; 2 Plant Conservation and Biology, Northwestern University, Evanston, IL 60203 USA; 3 German Centre for Integrative Biodiversity Research (iDiv) Halle-Jena-Leipzig, Puschstraße 4, 04103 Leipzig, Germany; 4 Biodiversity, Macroecology & Biogeography, Faculty for Forest Sciences & Forest Ecology, Goettingen University, Buesgenweg 1, 37077 Göttingen, Germany; 5 Department Community Ecology, Helmholtz Centre for Environmental Research-UFZ, Theodor-Lieser-Straße4, 06120 Halle (Saale), Germany; 6 Centre of Biodiversity and Sustainable Land Use (CBL), University of Goettingen, Büsgenweg 1, 37077 Göttingen, Germany; 7 Botanic Gardens Conservation International, U.S., 1151 Oxford Road, Botanical Division, San Marino, CA 91108 USA; 8 Campus-Institut Data Science, Göttingen, Germany; 9 Institute of Biology, Martin Luther University Halle-Wittenberg, Am Kirchtor 1, 06108 Halle (Saale), Germany

**Keywords:** Biodiversity, Conservation biology, Macroecology

## Abstract

Quantitative assessments of endemism, evolutionary distinctiveness and extinction threat underpin global conservation prioritization for well-studied taxa, such as birds, mammals, and amphibians. However, such information is unavailable for most of the world’s taxa. This is the case for the Orchidaceae, a hyperdiverse and cosmopolitan family with incomplete phylogenetic and threat information. To define conservation priorities, we present a framework based on phylogenetic and taxonomic measures of distinctiveness and rarity based on the number of regions and the area of occupancy. For 25,434 orchid species with distribution data (89.3% of the Orchidaceae), we identify the Neotropics as hotspots for richness, New Guinea as a hotspot for evolutionary distinctiveness, and several islands that contain many rare and distinct species. Orchids have a similar proportion of monotypic genera as other Angiosperms, however, more taxonomically distinct orchid species are found in a single region. We identify 278 species in need of immediate conservation actions and find that more than 70% of these do not currently have an IUCN conservation assessment and are not protected in ex-situ collections at Botanical Gardens. Our study highlights locations and orchid species in urgent need of conservation and demonstrates a framework that can be applied to other data-deficient taxa.

## Introduction

As human pressure on biodiversity mounts, there is an increasing need to improve allocation of scarce financial resources available for conservation^1^. Conservation prioritization is often focused on protecting: (1) Locations that have high species richness or are centres of endemism^1,2^, as investments in conservation in these areas are projected to have high rates of return^3^; (2) Evolutionarily distinct species, i.e. species that are on long branches in a phylogeny with no close relatives, as the extinction of these species would result in a disproportionate loss of the evolutionary history of Earth^4–6^; and (3) Globally-threatened species (the IUCN Red List of Threatened Species^7^ provides categorical assessments of the extinction risk of species based on internationally accepted criteria relevant to all species on Earth), as these species require conservation prioritization to prevent their extinction^8^.

Recent approaches combine these foci to set conservation priorities. The Evolutionary Distinctness and Globally Endangered (EDGE) and Evolutionary Distinctness Rarity (EDR) approaches consider evolutionary distinctness as well as global threat (IUCN Red List category) or rarity^4,9^. These integrated approaches have been used to prioritize global conservation efforts for amphibians^10^, mammals^11^, birds^12^, and Gymnosperms^13^. However, EDGE and EDR approaches are most useful in groups for which there is a high-resolution phylogeny and formal conservation assessments for most of the species. For example, of the 1090 species of Gymnosperms, 85% have been sequenced and 92% have IUCN Red List assessments^13^. Most taxa are not as thoroughly sequenced and assessed. For Angiosperms, only ~ 6.5% have an IUCN Red List assessment (www.redlist.org), and the phylogenetic resolution is still poor for many groups^14^. Here, we provide a framework for global conservation prioritization for taxa in which there is less information by synthesizing their global distribution and defining distinctiveness based on existing taxonomic and phylogenetic information.

Our framework is appropriate for large monophyletic groups such as the Orchidaceae, one of the most species-rich plant families on Earth, and known to be highly threatened by habitat loss and human trade^15–17^. Their international trade is so threatening that the Convention on Intentional Trade in Endangered Species of Wild Fauna and Flora lists all species in the Orchidaceae with an accepted species name^18^. Only 5.6% of orchid species with accepted names have been assessed by the IUCN Red List, and only 26% have regional or national assessments^19^. However, recent automated assessment of 13,000 orchid species using machine learning suggest that 31.2% of orchid species are possibly threatened with extinction^20^. Orchidaceae have a complex evolutionary history and biogeography, and there is not a consensus on the phylogenetic relationships of all species^14,21^, which further challenges species prioritization using the standard methods. Our framework takes a hybrid approach to prioritize species based on the information we do have about rarity (estimated range size and regional distribution, such as “botanical countries”) and distinctiveness (phylogenetic as well as taxonomic information on monotypic genera). Here, we aim to create manageable lists of priority species in several different ways and identify unique taxa across these lists that are in urgent need of conservation actions. We provide a description of these priority species and demonstrate that they are currently overlooked in global conservation efforts.

## Results and discussion

### Species richness

**Figure 1:**
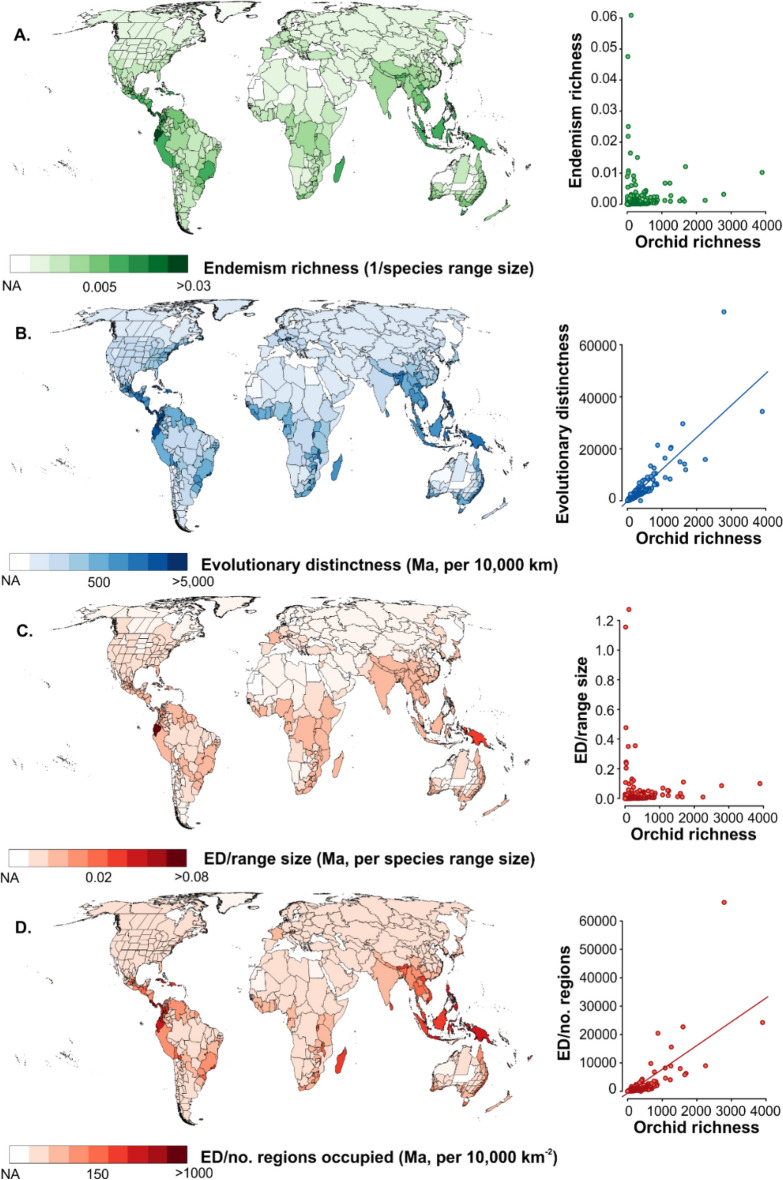
World maps highlighting centres of orchid (**A**) endemic species richness (**B**) evolutionary distinctness (**C**) evolutionary distinctness based on the range size of species (**D**) evolutionary distinctiveness based on the number of regions species occupy. All calculations, maps and other graphics were created using R 3.5.1, R Core Team. R software: Version 3.5.1. R Found. Stat. Comput. (2018) https://doi.org/10.1007/978-3-540-74686-7.

We quantify the distribution of 25,434 orchid species (89.3% of all orchid species with an accepted name and for which distribution information was available) across 495 mainland and island regions. Orchid species richness (corrected for area and then standardized to 10,000 km^2^) varies markedly among regions, in some cases by several orders of magnitude (Fig. 1A). Higher richness of orchids in tropical regions reflects the patterns observed for vascular plant species richness generally^22–24^. The Neotropics and South East Asia are a biodiversity hotspot for many taxa^2^, including an exceptional richness of orchid species. The high diversity of the Neotropics is thought to have resulted from the recent diversification of Neotropical species after the Andean uplift geological event^25^.

### Evolutionary distinctness

The evolutionary distinctiveness (ED) of a region, the sum of how phylogenetically distinct its species are based on the number of times each lineage has diverged^26^, is highly correlated with orchid richness (R^2^ = 0.87), and thus exhibits a similar global pattern (Supplementary Fig. S1). Ecuador has the highest predicted concentration of evolutionarily distinct taxa, representing over 24,000 Ma of dependent evolutionary history per 10,000 km^−2^. While we found that the highest predicted ED values were recorded for tropical mainland regions (following Ecuador—Thailand, Vietnam, Malaya, Costa Rica, Peru), ED was on average higher on islands (mean = 1597 Ma, Wilcoxon test *p* = 0.01). Among islands, continental islands displayed higher ED values than oceanic islands (mean = 2039 Ma, Wilcoxon test *p* = < 0.05), which is in line with previous observations that orchids generally are underrepresented on oceanic islands^27^. Nine percent of the total contribution of insular ED came from New Guinea alone, which may be a result of the movement of species out of the centre of origin in Australia into the topographic complexity of New Guinea^28^.

### Endemism

We quantify global patterns of endemic species richness in two ways. First, we considered the sum of ‘range size equivalents’ (hereafter RSE), the inverse of the number of regions occupied by a species^23^. Ecuador and New Guinea emerged as clear hotspots using this measure of endemism richness (per 10,000 km^2^) (Supplementary Fig. S2). Second, we considered range size as the total area of occupancy, which gives more weight species occupying small regions such as islands (e.g., *Dendrobium moorei* is endemic to Norfolk Island, which has a total area of only 63 km^2^). Using this measure, islands had higher endemic richness relative to mainland regions (Mean = 0.004 endemism richness, Wilcoxon test *p* = < 0.001), and endemism richness was greatest for the Caroline Islands and Norfolk Island Group (1D). For example, within the Caroline Islands, Palau has close to 100 species of orchids, many of which are endemic. This might be explained by its tropical climate and its proximity to New Guinea, a large orchid hotspot. Orchid species diversity on islands is known to be significantly lower than that of other large plant families^29^, however, our results show that certain archipelagos hold an extraordinary number of endemic species. True endemism is somewhere in between these two metrics we used, as one tends to give higher weight to large regions and the other to smaller regions. However, both methods found similar centres of orchid endemic richness in the Neotropics, southeast Asia, Madagascar, southern Australia and the southern cape of Africa (Fig. 1C, D).

### Evolutionary Distinctness Rarity (EDR)

Patterns of Evolutionary Distinctness Rarity (EDR) were calculated two ways: (1) ED and the rarity of a species measured as the inverse of the number of regions the species occupies (ED_Regions_corr_), and (2) taxa. When numbers of regions were considered, hot spots for EDR are in the Andes and in Southeast Asia (Fig. 1B), whereas when cumulative area of regions was considered, hotspots include islands such as New Guinea, Madagascar and Micronesia (Fig. 1C).

### Monotypic taxa

**Figure 2:**
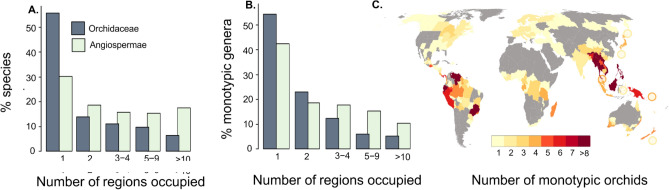
(**A**) Histogram showing the proportion of orchids and Angiosperm species (excluding orchids) that are located in a given number of geographic regions. (**B**) Histogram showing the proportion of monotypic orchid genera that are located in a given number of geographic regions. (**C**) World map highlighting centres of monotypic orchid species richness. All calculations, maps and other graphics were created using R 3.5.1, R Core Team. R software: Version 3.5.1. R Found. Stat. Comput. (2018) https://doi.org/10.1007/978-3-540-74686-7.

Monotypic taxa are often recognized by conservationists as evolutionarily distinct, and are therefore worthy of special consideration^26,30^. As with other Angiosperms, approximately 14.8% of orchid genera are monotypic (113/761 genera)^31^. However, 53% percent of all orchid species occur in just one region, compared to 31% of other types of Angiosperms (Fig. 2A). When combining this information, a high proportion (57%) of orchid monotypic taxa occur in only one region (Fig. 2B). Southeast Asia and the Neotropics have the highest numbers of monotypic taxa (Fig. 2C).

### Priority lists

**Figure 3:**
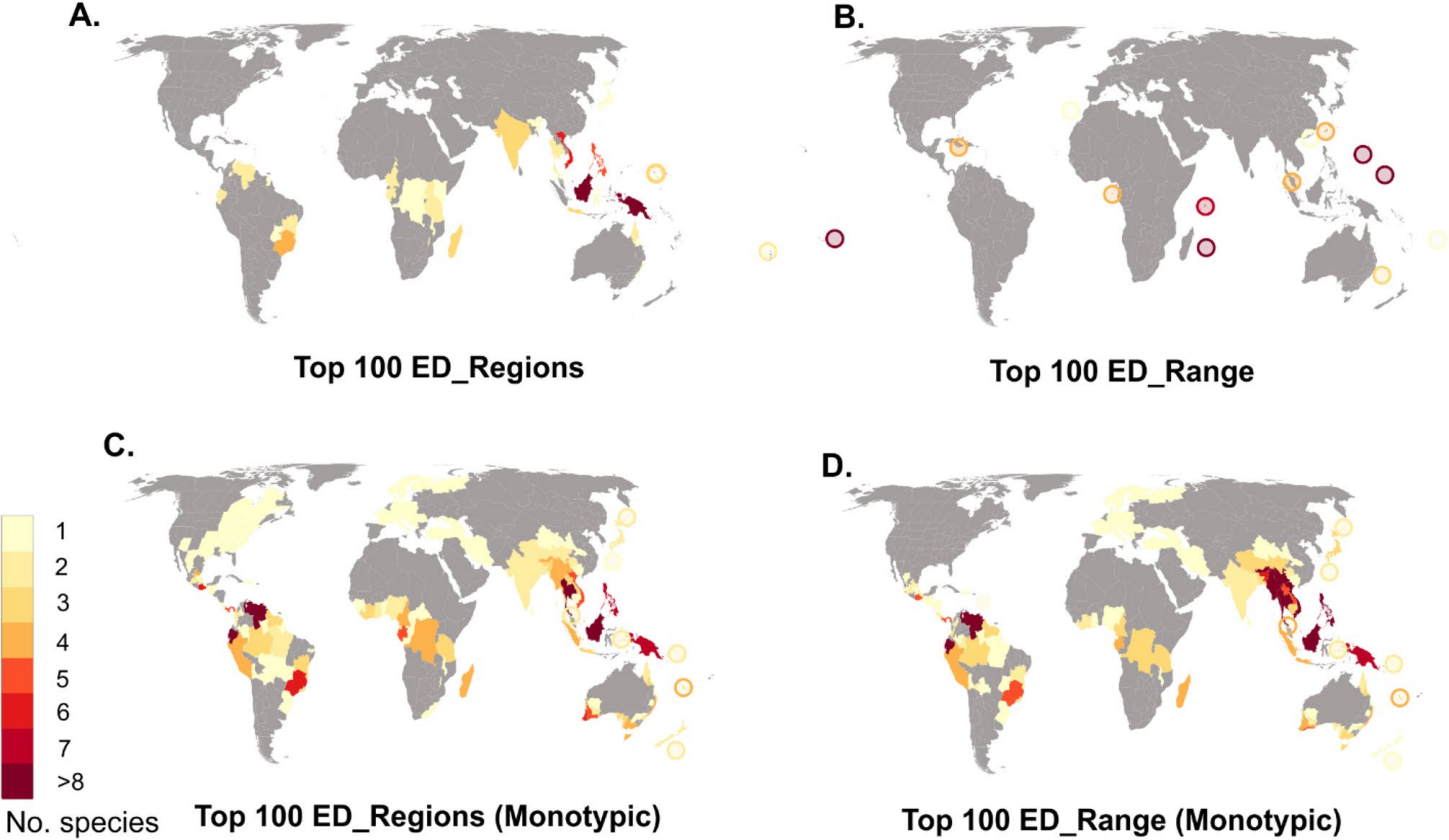
World maps highlighting centres of priority species based on several metric scores: (**A**) evolutionary distinctness rarity based on the number of regions species occupy (ED_Regions), (**B**) evolutionary distinctness rarity based on the range size of species (ED_Range), (**C**) monotypic genera and rarity based on the number of regions species occupy, and (**D**) monotypic genera and rarity based on the range size of species. All calculations, maps and other graphics were created using R 3.5.1, R Core Team. R software: Version 3.5.1. R Found. Stat. Comput. (2018) https://doi.org/10.1007/978-3-540-74686-7.

We identify those orchid species that should have highest conservation priority by presenting the top 100 species from each of four approaches: (1) highest ‘ED_Regions’ metric scores, (2) highest ‘ED_Range’ metric scores, (3) monotypic orchids with the highest ‘ED_Regions’ metric scores, and (4) monotypic orchids with the highest ‘ED_Range’ metric scores. These approaches resulted in different species lists, with species concentrated in different locations, for example, methods based on regions identified species in New Guinea and Malesia as highest priority, while those based on range prioritized species on small islands (Fig. 3). When combined, we identified 278 unique taxa, with 63 species identified by two of the approaches, and 26 by three of the approaches. *Dilochiopsis scortechinii*, an epiphytic orchid found in Malesia, was identified as a taxon in need of conservation action by all four approaches.

While 41% (113) of the species on our final list are from monotypic genera, only 20% (32) of the species prioritized based on phylogenetic methods belonged to monotypic genera. This is likely because monotypic taxa have not been appropriately assessed phylogenetically (35% have no information on GenBank). Conversely, 38% of the priority taxa are represented by multiple species from the same genus. Large genera such as *Dendrobium* (1800 species) and *Bulbophyllum* (2000 species) have 20 and 15 species on our list, respectively, and were overrepresented based on their genera size on the ED range list (Supplementary Fig. S3). Many of these species are endemic to small islands and, while known to not be distinct due within their large clade, have no information on GenBank that can precisely indicate their evolutionary distinctiveness (e.g., *Dendrobium adamsii*, endemic to the island of Pohnpei).

Generally, application of EDR rankings are used to provide lists of species that should be considered priorities for conservation action as they represent species that hold distinct and important evolutionary information and are also exceedingly rare^9^. Our work acknowledges that the information we have on phylogeny, taxonomy and species distributions is incomplete. However, by calculating EDR rankings in different ways, we aim to capture the species that are in the most urgent need for conservation actions.

### Description of the priority species

**Figure 4:**
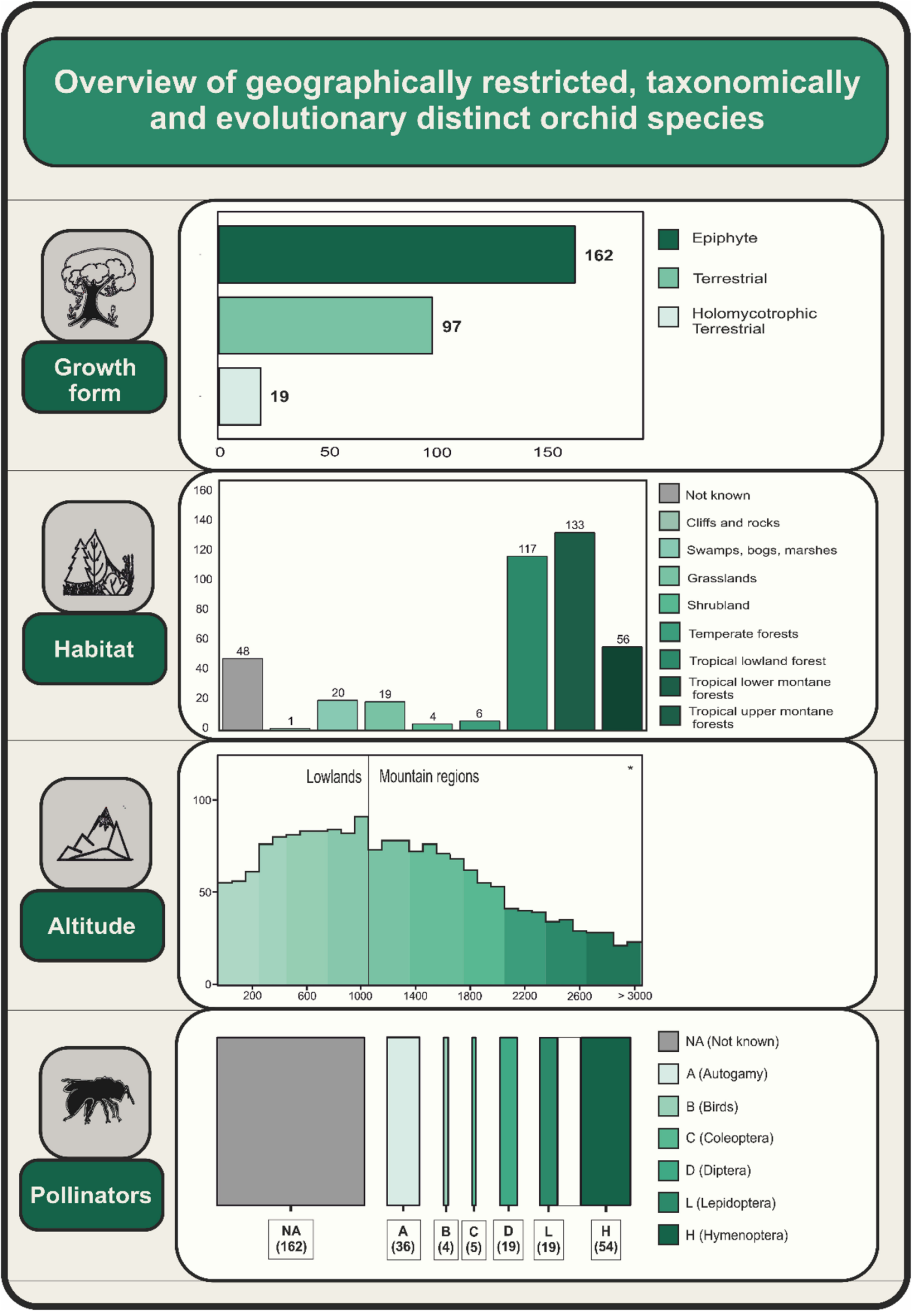
Overview of the 278 priority orchid species, including growth form, habitat, altitude, and pollination syndrome.

Our compiled priority species list is primarily comprised of epiphytic orchids that occur in tropical forests at middle to high altitudes (Fig. 4). This is an unsurprising result, as it reflects the predominant growth form, habitat associations and distribution of the Orchidaceae family as a whole^21,32^. Interestingly, although most orchid species are mixotrophic, obtaining carbon resources both via photosynthesis and from a fungal symbiont, our list contains a several mycoheterotrophic species, which depend entirely upon the mycorrhizal network for carbon. Such species present an increased challenge for ex situ conservation. Many of the priority species are found in mountain regions and montane forest habitats (Fig. 4), which are especially threatened by climate change. In addition, many priority species live in regions with some of the highest rates of deforestation (Supplementary Fig. S4)^33^. Further complicating conservation efforts, the basic pollination ecology is unknown for more than half of our priority species (Fig. 4). For those with known or inferred pollinator syndromes, a high proportion are bee pollinated and several are autogamous (Fig. 4). Orchids are notoriously specialized in their pollination biology^34^, and are one of the most pollen limited plant families in the world^35^. The relative lack of information on the pollination and other ecological requirements of these priority species highlights the need for more basic research on all monotypic orchids and an assessment of how pollination syndromes are conserved across the orchid phylogeny.

### Actions to be taken

**Figure 5:**
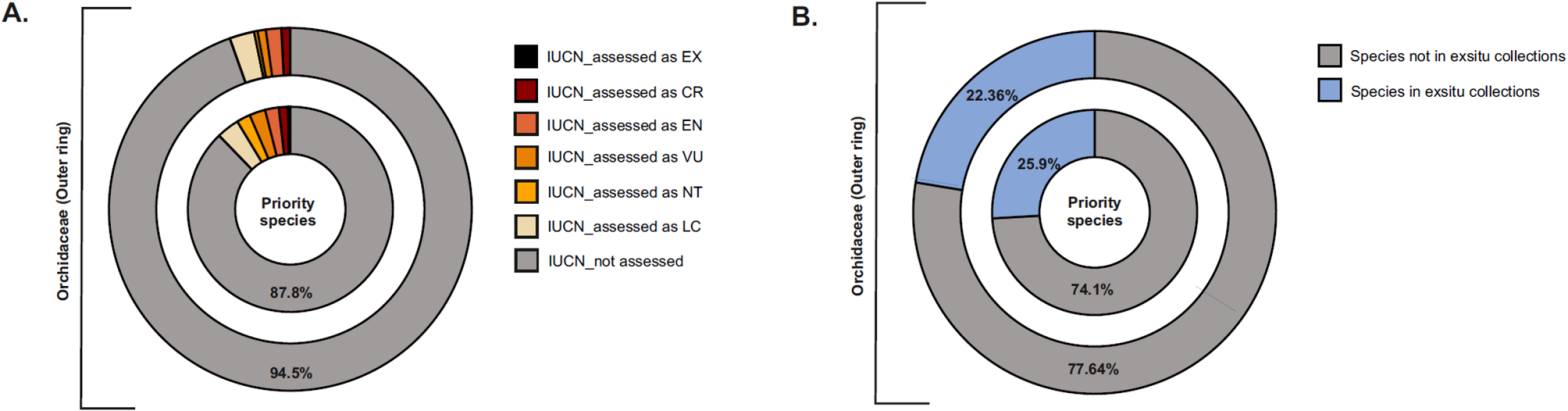
(**A**) Proportion of orchid species with IUCN assessments. (**B**) Proportion of orchid species found in botanic garden collections globally.

Our analysis provides identifies species needing immediate conservation action within this diverse plant family. However, we only assess here rarity based on the number of regions and on the area of occupancy of species, and do not incorporate information on species local abundances. An immediate next step should be to ensure that all species identified here as having a high priority for conservation are formally assessed under the IUCN red list assessment. Indeed, the Global Strategy for Plant Conservation, part of the Convention on Biological Diversity, set a goal of assessing all known vascular plant species by the end of this year^36^. However, currently only 37 of our 278 priority species (13.3%) have an IUCN red list assessment (Fig. 5). Of those few assessed, most are assessed as threatened by extinction (Critically Endangered, Endangered or Vulnerable), as would be expected. However, 11 species are designated as Least Concern. Six of these are species belonging to monotypic genera and made the top 100 list even though they occur in more than one region. For example, *Chamorchis alpina*, Europe’s smallest orchid, is on the priority list; this species is highly distinct but is comparatively widespread in the mountain regions of several European countries and locally abundant in some of those locations. There is an urgent need for IUCN red listing of the Orchidaceae plant family as well as for land plants as a whole^19,37^, and new methods of automated assessments might provide a pathway to achieve this global goal^20^. However, such assessments require information about the current occurrences of all species, which would be greatly facilitated by more research activity in regions with high richness and endemism of orchids. The lack of distribution data limits the potential of using these priority lists to determine if existing protected areas are sufficient to ensure in situ conservation of these species.

Thus, safeguarding these priority species in ex situ collections at botanical gardens, either as living plant materials or in seed bank collections, is critical to ensuring that their extinctions from the wild are not also extinctions from our planet^38^. Indeed, the Global Strategy for Plant Conservation set a target for 2020 that “at least 75 per cent of threatened plant species are represented in ex situ collections, preferably in the country of origin. Further, at least 20 percent of these species should be available for use in recovery and restoration programs.” Only 72 of our 278 priority species (25.9%) are currently present in ex situ collections at botanical gardens, and of these, 31 are found in a single collection (Fig. 5). There are many challenges to building ex situ collections for orchids, including identifying the species with the most need in this large plant family, the difficulty of finding species in the wild and gaining a collecting permit, and the difficulty in germinating and growing some species of terrestrial orchids in ex situ conditions.

Even though we might lack specific knowledge of many important taxa including pollinators and fungal partners, there is no technical reason for any of them to go extinct^39^. There are a multitude of tools available to undertake the safe-guarding of plant species^16,37^. These include ensuring that a genetically appropriate population of each species is represented in living collections in botanic gardens and undertaking captive breeding across institutions where these species are held^40^. Ex situ conservation and breeding of priority orchids is ideally undertaken by conservation professionals at institutions capable of ensure the integrity of the provenance of species and ensuring the long-term specialized care these species require. The global botanic garden community is particularly well suited in this regard^38,39^. Priority species that are difficult to germinate and grow should be funding priorities for horticultural research. We urge researchers at these gardens, as well as private individuals with diverse collections, to contact us to determine if you hold species coincident with our priority orchid list. If so, we encourage private collectors to consider donating plant or reproductive materials such as pollen or seeds to a botanical garden. Another way that private collections can become part of a broader conservation solution is to register taxa with the Botanic Gardens Conservational International’s PlantSearch database^41^. This database accommodates private collections, in addition to botanical garden collections, and connects these collections to thousands of requests for information and material each year.

## Conclusion

Our global synthesis reveals the places on Earth that are in urgent need for conservation prioritization to protect Orchidaceae biodiversity and evolutionary history. In addition, we identify 278 unique species that should be prioritized because they have restricted distributions and represent either unique evolutionary lineages and/or have unique morphological features. The Orchidaceae is a beloved and charismatic plant family, and there are active groups of academic and industry scientists as well as private collectors dedicated to understanding and conserving them. Despite this, there are significant knowledge gaps in their distribution, natural history, taxonomy, and molecular phylogeny, which make prioritization difficult. The approach that we have taken to provide a priority scheme for conservation in the presence of incomplete information should serve as a model for other large taxonomic groups, including those that are less charismatic and will therefore have even more incomplete information. Preliminary prioritizations such as this one are valuable to conservation and also provide quantitative assessments of future research needs.

## Methods

### Species lists and distribution data

We compiled a list of Orchid species from the Global Inventory of Floras and Traits database (GIFT, http://gift.uni-goettingen.de/home)^42^, a digital repository of floras and checklists from around the world^42^. In addition to checklists for smaller geographical regions including many islands, the GIFT database has orchid species lists at coarser grain sizes like botanical countries and political units, most of which are derived from the World Checklist of Selected Plant Families (WCSP, http://wcsp.science.kew.org/). In order to gain complete global coverage of orchid distributions, we extracted species lists for all smaller geographic regions first, retaining those that overlapped with larger regions if they collectively made up a larger region. For example, we would prefer a state or province over an entire botanical country if data were available at the finer scale. For countries where we do not have complete regional checklists (e.g., India), we used botanical country-level checklists derived from the WCSP. Because of the differences in geological history, size, and processes at which they gain species, islands were categorized as continental and oceanic origin. To reduce potential bias when comparing islands to larger mainland regions, we grouped all single islands into their immediate archipelagos when possible (e.g., La Réunion and Mauritius = Mascarene Islands) and omitted any remaining single islands < 50 km^−2^ from further consideration. Our final distribution dataset included 25,434 orchid species (89.9% of 28,484 total global accepted orchid species) distributed across 495 mainland and island sites.

### Taxonomic considerations

Although we have near complete distribution data in GIFT for the Orchidaceae, it wasn’t possible to match all species to the Smith and Brown phylogeny^14^, despite the phylogeny containing information for > 30,000 orchids. This is largely due to taxonomic issues. For one, we did not consider hybrids, variants, or those abbreviated with *conferatur* (cf.) and *affinis* (aff.) in our analyses, nor did we accept morphospecies, which account for 1991 species in the phylogeny. Additionally, most species names in the GIFT database are standardized according to The Plant List^43^, which is not the most up to date taxonomical resource for the Orchidaceae. In our preliminary effort to standardize species names from the phylogeny according to TPL, we found that 915 species names were synonyms, 189 names were unresolved, and 1894 names were not known to TPL. We therefore opted to refer to the World Checklist of Selected Plant Families^31^, which has taxonomically revised the Orchidaceae more recently, to determine the taxonomic status and distributions of all unresolved and unrecognized taxa on the phylogeny. In total, we could match the phylogeny with 22,702 orchid species for which we have the most complete distribution information (79.7%). It is important to note, however, that true evolutionary distinctness may be under-represented in some regions. Out of the species that could not be matched, 58% were island dwellers. Islands that were most affected include New Guinea (312 species unmatched), Philippines (121 species), Sumatra (104 species), and Taiwan (94 species), while Peru (316 species), Costa Rica (183 species), Guatemala (156 species), and Vietnam (138 species) were the most affected mainland regions. These regions are relatively diverse in orchids, which may explain the difficulties in obtaining genetic material to cover all species. Similarly, some genera are over-represented (e.g., *Bulbophyllum*, *Dendrobium*, *Epidendrum*, *Habenaria*, and *Maxillaria*) because of their large contribution to overall orchid diversity.

### Orchid species richness and endemism (rarity)

Orchid species richness was calculated simply as the number of species within a region. However, for a better comparison of species richness among islands and mainland regions, some of which vary dramatically in size, we modified the classical species-area model to correct for area effects:

$$S=c{A}^{z},$$

where* S* (defined below as *S*_*obs*_) denotes observed species richness, *A* is the total area of a region,* c* is a model constant, and *z* is the slope of the species-area relationship in log–log space. Here, we define the constant *c* as area-corrected species richness *S*_*corr*_, which can be solved by modifying the species-area model as follows:

$${S}_{corr}={\left(\frac{{S}_{obs}}{A}\right)}^{z}.$$

Because islands and mainland regions gain new species at different rates^44^, we derived standardized *z*-scores for orchid-area relationships on islands separately from mainland regions, making a further distinction between continental and oceanic islands. Finally, we standardized the species richness of each region to 10,000 km^2^ to allow for a better comparison between island and mainland regions following^23^, giving a final equation of:

$${S}_{corr}={S}_{obs}{\left(\frac{\mathrm{10,000}}{A}\right)}^{z}.$$

Unlike species richness, the concept and definition of endemism is not so straightforward. In most cases, a species is considered endemic if it is restricted in range size or to a single region^45^. We therefore opted to compare two different endemism metrics that will ultimately lead to two different measures of evolutionary distinctness rarity. First, we considered a species ‘range size equivalent’ (hereafter RSE), which is calculated by taking the inverse of the number of regions occupied by a species^23^. A species found in a single region will therefore have a value of 1, while a species that occupies two regions will have a value of 0.5, and so on. One major limitation of this method specific to our dataset is that we do not predict species occurrences in equal-area grid cells. Thus, a species that is restricted to India would be considered ‘rare’, despite India encompassing an area of 3.28 million km^2^. Conversely, a species restricted to Bolivia would not be considered ‘rare’ if it occurs in all 9 primary sub-divisions. To combat this bias, we followed the same method used to standardize species richness and correct for area. For our second measure of orchid rarity, we considered the metric of ‘endemism richness’, also termed ‘weighted endemism’. Endemism richness was calculated by taking the inverse of a species range size, which was then summed for each region following Kier et al.^23^. Unlike other endemism metrics, endemism richness assigns weights to species according to their total area of occupancy (range size), assigning higher values to species with smaller range sizes (e.g. an endemic to a small oceanic island), and lower values to species with larger range sizes (e.g. species with a cosmopolitan distribution covering large geographical areas^46^. Based on this method, widespread species, like *Dactylorhiza viridis*, which occupies 230 different geographical regions totaling 60,791,223 km^2^ in area would be assigned very low endemism richness values, while endemic species like *Dendrobium moorei* of Norfolk Island, which occupies a total area of 63 km^2^ would be weighted higher. Because endemism richness directly considers a species range size, we did not need to standardize the summed values for each region.

### Evolutionary distinctness rarity

The Evolutionary Distinctness Rarity (EDR) score of a species is composed of two attributes; (1) evolutionary distinctness (ED), which is a measure of how phylogenetically distinct a species is and which accounts for the number of times a lineage has diverged^4^, and (2) rarity, measured here in terms of either (a) a species’ geographical range size, or (b) the number of regions occupied. For the calculation of ED, we used the publicly available mega-phylogeny of seed plants^14^, subset for the Orchidaceae. Evolutionary distinctness was then quantified using the ‘equal splits’ approach^47^, which equally divides evolutionary time (branch length) among daughter branches, the sum of which is equal to the clade’s total evolutionary history. Thus, species derived from multiple descendants will have a low ED score, while those that have not diverged throughout the clades evolutionary history will maintain the highest ED scores. Each orchid species’ ED score was then divided by (a) their geographical range size and (b) the total number of regions they occupied to obtain two different EDR metrics; ‘ED_Range’ and ‘ED_Regions’, which were then summed for each geographical region. Because a region’s ED_Range score already takes into account species geographical range sizes, and thus total area of occupancy, we did not standardize these scores according to area, although it was necessary to standardize and area-correct ED_Regions. All calculations, maps and other graphics were created using R 3.5.1^48^ using the packages ‘ape’^49^, ‘phytools’^50^, ‘phylobase’^51^, ‘rgdal’^52^, ‘reshape’^53^, and ‘taxonstand’^54^.

### Taxonomic distinctness rarity

We defined species to be taxonomically distinct when they are the sole representatives of their genus. A list of potentially monotypic genera was extracted from TPL, with each entry being subsequently checked against the records in the WCSP. The rarity of each species was assessed by taking into consideration both the number of regions in which the taxon has been recorded and its total area of occupancy, following the above-mentioned methodologies. Species occurring in just one or two regions were considered to be rare. We then compared the proportion of monotypic genera and the proportion of monotypic genera in a given number of geographical regions between orchids and all Angiosperms (210,576 Angiosperm species, excluding orchids). While we acknowledge that the status of some monotypic genera is likely to change with future phylogenetic analyses and taxonomic research, we aim to present the most up to date assessment of monotypic orchid genera available from the WCSP. The number of monotypic orchids were aggregated for each region and mapped to highlight hotspots.

### Priority lists

We identified those orchid species that should be considered the highest conservation priority by presenting the top 100 species from each of four approaches: (1) highest ‘ED_Range’ metric scores, (2) highest ‘ED_Regions’ metric scores, (3) monotypic orchids with the highest ‘ED_Range’ me tric scores, and (4) monotypic orchids with the highest ‘ED_Regions’ metric scores. A complete list of all species identified using these four approaches is available by request from the corresponding author to protect these species from over-collection.

### Description of priority species

We provide an overview of the ecology of the priority species, including growth form, habitat type, altitudinal range and pollination biology.

#### Growth form

The WCSP was used in order to retrieve information about the life form of all species within our summarized prioritization list. Life form categories used by the WCSP were synthesized into three major categories: epiphytes, terrestrials and holomycotrophic terrestrials based on their growth form and obligate association with mycorrhizal fungi^16,55^.

#### Habitat type and altitudinal range

We relied on the collective volumes of the *Genera Orchidacearum*^56–60^ and a variety of online databases, identification keys and original species descriptions to compile information on the preferred habitat types and altitudinal ranges of the priority species. Habitat types were summarized into eight broad categories, including species for which habitat preference is unknown: coastal habitats (including mangrove forests), wetlands (including swamps, bogs and marshes), grasslands, shrublands, temperate forests (including sclerophyllous dry forests, deciduous broadleaf forests, evergreen needleleaf forests, broadleaf evergreen forest), tropical lowland forests, and montane forests (including evergreen broadleaf rain forests, semi-evergreen moist broadleaf forests, deciduous/semi-deciduous broadleaf forests, sclerophyllous dry forests, needleleaf forest and mixed needleleaf/broadleaf forests).

#### Pollination biology

For some priority orchid species, detailed information on the pollination biology was available in the primary literature, or is inferred by experts based on floral traits^56–61^. We used the information available to create six broad categories to classify pollination syndrome: unknown, autogamy, bird-, hymenopteran-, dipteran-, coleopteran- or lepidoptera-pollinated.

### Assessed conservation status

In order to determine the conservation status of our priority species, we cross referenced the accepted name of each unique taxon against the current IUCN Red List^7^, which represents the global threat assessment for each species. We did not cross reference our priority species with national, regional or other assessments because they may not be conducted with the same uniform standards as the IUCN Red List. We estimated the proportion of species for which a Red List assessment was available by the most recent IUCN assessment and by the 1997 assessment. We considered the latter to include species for which the extinction risk has not yet been reassessed. The conservation status of the priority species is compared to the Orchidaceae as a whole.

### Presence of priority species in ex situ collections

To assess the current ex situ representation of the priority species identified above, we used BGCI’s PlantSearch^41^, the only global database of plants, seeds, and tissues maintained in over 1100 living botanical collections around the world. We matched the priority species with PlantSearch records using genera and species epithets and calculated the total numbers of living plant and seed collections recorded in PlantSearch that reported each species.

## Supplementary Information

Supplementary Figures.

## Data Availability

Data underpinning these analyses are available openly (IUCN Red List assessment www.redlist.org, Global Inventory of Floras and Traits database GIFT, http://gift.uni-goettingen.de/home, and the World Checklist of Selected Plant Families WCSP, http://wcsp.science.kew.org/) and upon request (information on ex situ conservation status, BGCI’s PlantSearch). Given the extraordinary threat of harvesting of the priority orchid species identified in this manuscript, we will make data available on the identity and features of these species upon the corresponding author's request.
