# Supplementary material for: Global conservation prioritization for the Orchidaceae
Source: Sci Rep. 2023 Apr 25;13:6718. doi: 10.1038/s41598-023-30177-y (PMC10130154; doi:10.1038/s41598-023-30177-y)
Supplement: Supplementary file 1 — Supplementary Figures. [file 41598_2023_30177_MOESM1_ESM.docx]

**
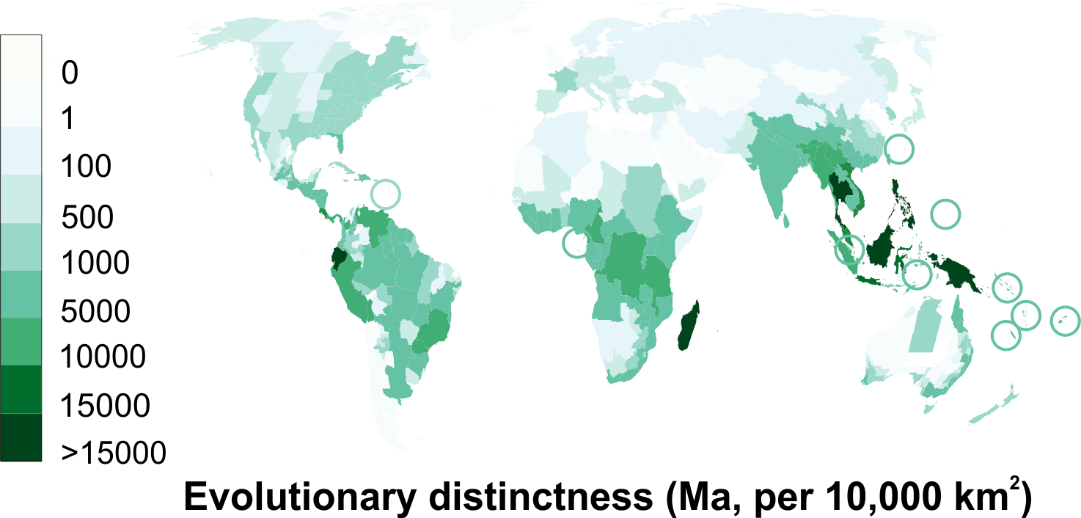
**

Figure S1. World map highlighting centres of orchid evolutionary distinctiveness. Evolutionary distinctiveness of a region is the sum of how phylogenetically distinct its species are based on the number of times each lineage has diverged. All calculations, maps and other graphics were created using R 3.5.1, R Core Team. R software: Version 3.5.1. R Found. Stat. Comput. (2018) doi:10.1007/978-3-540-74686-7


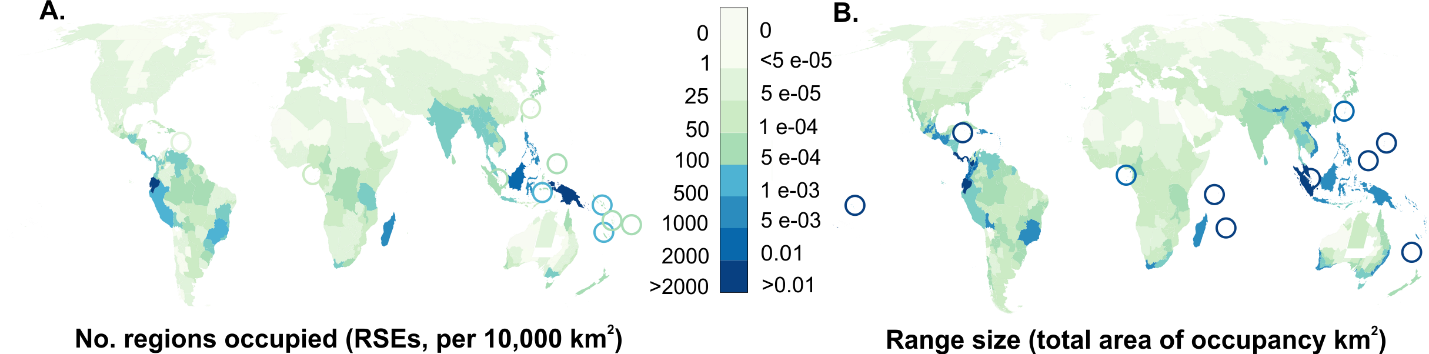


Figure S2. World map highlighting centres of orchid endemic species richness. Endemic species richness is calculated as: A) as the inverse of the number of regions occupied by a species and B) the inverse of the range size (total area of occupancy). All calculations, maps and other graphics were created using R 3.5.1, R Core Team. R software: Version 3.5.1. R Found. Stat. Comput. (2018) doi:10.1007/978-3-540-74686-7

A.


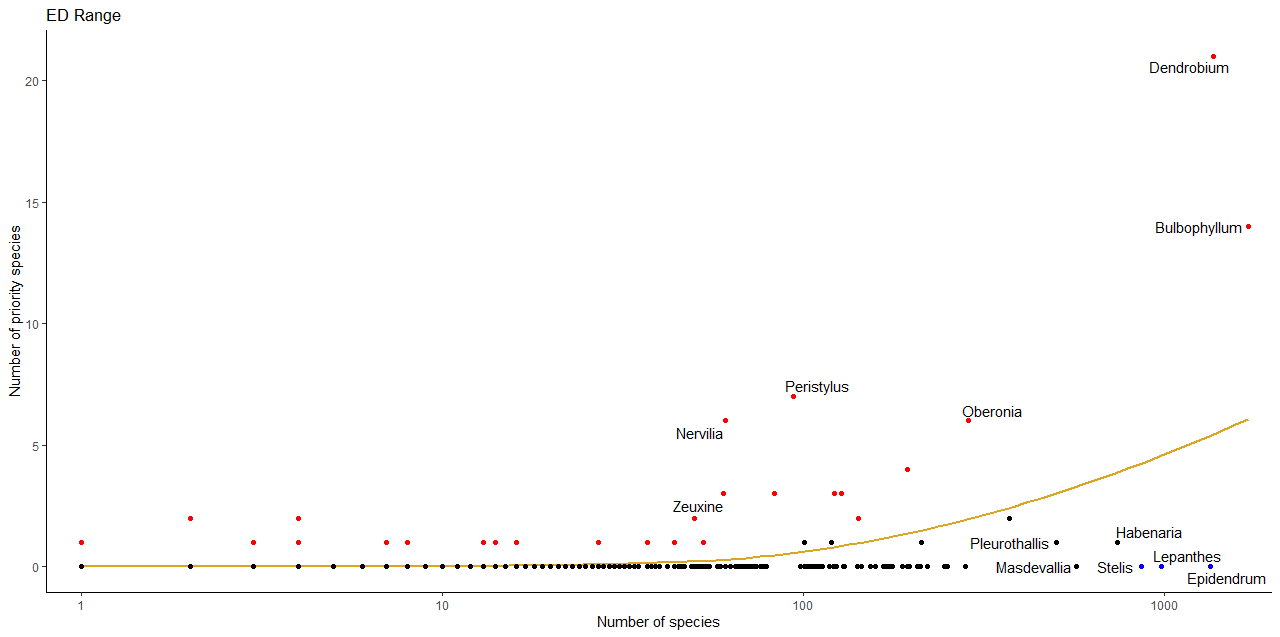


b.
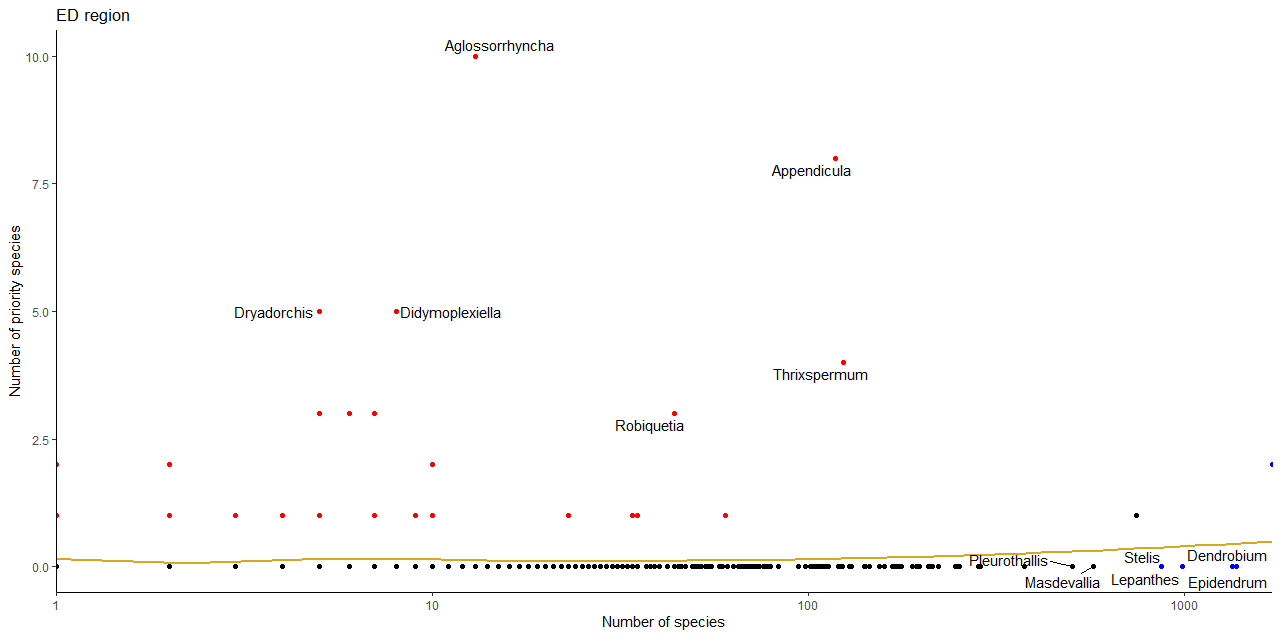


Figure S3. We tested whether each genus was over-represented (red points) or under-represented (blue points) in the priority list for a) ED range and b) ED region using the phyper function in R. The 12 genera with names in the figure are those with the highest deviation from a loess type model (six overrepresented, six underrepresented).


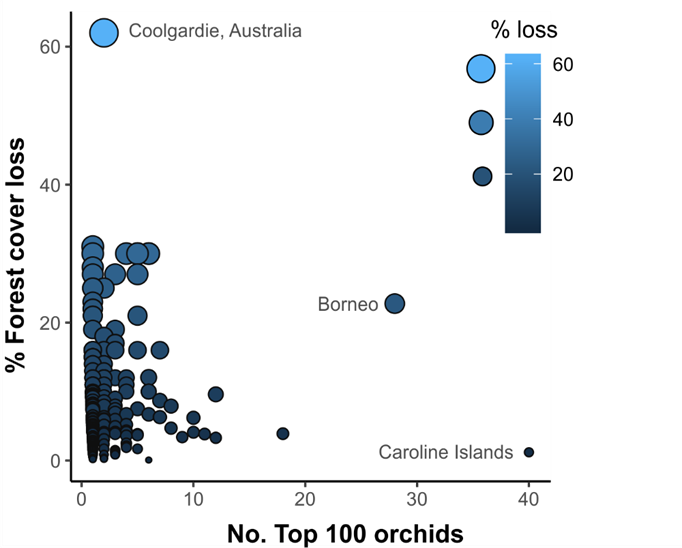


Figure S4. Plot showing 197 regions containing ‘Top 100 orchids’ and their % forest cover loss from 2001 to 2018 derived from the Global Forest Watch (GFW). Regions with <0.01% of forest cover since 2010 were excluded.

Citation: Global Forest Watch. 2014. World Resources Institute. Accessed on (28.11.2019). www.globalforestwatch.org.
